# Construction of a nomogram to predict the survival of metastatic gastric cancer patients that received immunotherapy

**DOI:** 10.3389/fimmu.2022.950868

**Published:** 2022-09-26

**Authors:** Miaomiao Gou, Niansong Qian, Yong Zhang, Lihui Wei, Qihuang Fan, Zhikuan Wang, Guanghai Dai

**Affiliations:** ^1^ Medical Oncology Department, The Fifth Medical Center, Chinese People’s Liberation Army General Hospital, Beijing, China; ^2^ Medical Oncology Department, Hainan Medical Center, Chinese People’s Liberation Army General Hospital, Beijing, China; ^3^ Medical Oncology Department, The Second Medical Center, Chinese People’s Liberation Army General Hospital, Beijing, China; ^4^ Department of Medicine, Genetron Health (Beijing) Co. Ltd., Beijing, China

**Keywords:** nomograms, metastatic gastric cancer, immunotherapy, inflammation markers, predicting

## Abstract

**Background:**

Immunotherapy has shown promising results for metastatic gastric cancer (MGC) patients. Nevertheless, not all patients can benefit from anti-PD-1 treatment. Thus, this study aimed to develop and validate a prognostic nomogram for MGC patients that received immunotherapy.

**Methods:**

Herein, MGC patients treated with anti-PD-1 between 1 October 2016 and 1 June 2022 at two separate Chinese PLA General Hospital centers were enrolled and randomly divided into training and validation sets (186 and 80 patients, respectively). The nomogram was constructed based on a multivariable Cox model using baseline variables from the training cohort. Its predictive accuracy was validated by the validation set. The consistency index (C-index) and calibration plots were used to evaluate the discriminative ability and accuracy of the nomogram. The net benefit of the nomogram was evaluated using decision curve analysis (DCA). Finally, we stratified patients by median total nomogram scores and performed Kaplan–Meier survival analyses.

**Results:**

We developed the nomogram based on the multivariate analysis of the training cohort, including four parameters: surgery history, treatment line, lung immune prognostic index (LIPI), and platelet-to-lymphocyte ratio (PLR). The C-index of the nomogram was 0.745 in the training set. The calibration curve for 1- and 2-year survival showed good agreement between nomogram predictions and actual observations. In the validation group, the calibration curves demonstrated good performance of the nomogram, with a C-index for overall survival (OS) prediction of 0.713. The OS of patients with a score greater than the median nomogram score was significantly longer than patients with a score lower or equal to the median (*p* < 0.001).

**Conclusion:**

We constructed a nomogram to predict the outcomes of MGC patients that received immunotherapy. This nomogram might facilitate individualized survival predictions and be helpful during clinical decision-making for MGC patients under anti-PD-1 therapy.

## Introduction

Gastric cancer (GC) is the third most common cancer and the third leading cause of cancer deaths in China (1). Its incidence and mortality rates are the highest in Eastern Asia (2), and half of GCs occur with metastases (3). In the chemotherapy era, the treatment of metastatic GC (MGC) mainly comprehends fluoropyrimidine and platinum or docetaxel-based therapy, presenting survival of around 1 year (4, 5). With the advent of immunotherapy, immune checkpoint inhibitors (ICIs) have shown promising anticancer activity for many cancers (6–8), including MGC (9–12). Therefore, fundamental changes have been made to treat MGC, especially in the first or third lines, with programmed death-1 (PD-1) inhibitor, a type of ICI, which prolonged the overall survival (OS) to more than one and a half years.

However, the effects of immunotherapy vary among MGC patients. Thus, evaluating the prognosis of patients under different conditions is essential for medical care and patient stratification. Previous studies have demonstrated the efficacy of prognostic models in predicting the OS of GC patients exposed to chemotherapy based on clinicopathological factors (13, 14). Although programmed death-ligand 1 (PD-L1) expression, tumor mutation burden (TMB), and microsatellite instability (MSI) are the most common biomarkers to predict which patient might benefit from anti-PD-1 treatment, some of those markers remain controversial. The system inflammation index (SII) in peripheral blood has also been a valuable prognostic factor (15–17). The recent studies of our research group have demonstrated that the hemoglobin (Hb) level and the neutrophil-to-lymphocyte ratio (NLR) in peripheral blood might be associated with the survival outcomes of patients receiving PD-1 inhibitors (18, 19).

Nomograms are commonly used to predict survival rates with high accuracy compared to prognostic grouping or scoring systems (20). Several cancers, including prostate (21), colon (22), and breast (20) cancers, and intrahepatic cholangiocarcinoma (23) have well-constructed nomogram-based prognostic models. Regarding GC, it has been demonstrated that nomograms can predict the survival of MGC patients who received a combination of cytotoxic chemotherapy as first−line treatment (24) or patients with localized GC after curative resection (25). Few nomograms have been reported for MGC patients treated with PD-1-based therapy. Therefore, this study aimed to create and validate a more comprehensive nomogram combining clinicopathological variables and blood indexes to predict the 1- and 2-year survival of MGC patients under immunotherapy. Our current results might provide a more personalized and comprehensive information outlook for clinicians after initiating immunotherapy.

## Materials and methods

### Study patients

Herein, MGC patients who underwent immunotherapy between 1 October 2016 and 1 June 2022 at two Chinese PLA General Hospital sites were retrospectively enrolled. Patients were eligible if they meet the following criteria: 1) histopathological confirmation of late-stage GC, 2) received at least two anti-PD-1 treatment cycles, 3) complete availability of clinical demographic features and serum tumor markers, 4) a week of complete blood test before the anti-PD-1 treatment, and 5) survival data from the beginning of anti-PD-1 treatment to death. Patients who did not present at least one clinicopathological variable of interest (age, gender, histological differentiation, performance status, prior gastrectomy, metastatic sites, initial laboratory values, treatment regimen, and survival outcomes) were excluded. According to these criteria, 266 patients were initially selected for model development. They were randomly assigned to the training set (approximately 70%; *n* = 186) to establish the prognostic model and to the validation set (remaining 30%; *n* = 80). The Ethical Committee of the Chinese PLA General Hospital granted ethical approval for this observational retrospective research, and all patients provided written informed consent.

The following demographic, clinical, and pathological data were collected: gender, age, Eastern Cooperative Oncology Group performance score (ECOG PS), primary tumor site, surgery history, presence of liver metastasis, number of metastatic organs, anti-PD treatment line, and histological differentiation. PD-L1 expression data were also collected. The expression of PD-L1 was analyzed on tumor and tumor-associated immune cells by immunohistochemistry [combined positive score (CPS)] according to the standard practice for each center. Positive expression was characterized by at least one CPS.

Additionally, routine blood parameters, including absolute white cell count, neutrophil count, absolute platelet count, lymphocyte count, lactate dehydrogenase (LDH), hemoglobin (HB) levels, tumor marker carbohydrate antigen 19-9 (CA 19-9), and carcinoembryonic antigen (CEA) at baseline before anti-PD-1 treatment (within 7 days before the first treatment), were extracted from electronic medical records. Peripheral inflammatory indexes were also measured, including NLR, platelet-to-lymphocyte ratio (PLR), monocyte-to-lymphocyte ratio (MLR), and dNLR [absolute neutrophil count/(white blood cell count − absolute neutrophil count)]. The best cutoff values for NLR, PLR, MLR, and dNLR were determined by receiver operating characteristic (ROC) analyses. The lung immune prognostic index (LIPI) was calculated based on the dNLR and LDH values. The good LIPI group was defined by dNLR < candidate cutoff value and LDH levels below the upper limit of normal (ULN); intermediate or poor LIPI was defined by dNLR ≥ candidate cutoff value or LDH levels over the ULN.

### Immunotherapy

The PD-1 inhibitors given as a first-line regimen and receipt of second-line or later chemotherapy were identified. PD-1 inhibitors with chemotherapy were mainly used in the first line; PD-1 inhibitor monotherapy/PD-1 inhibitors plus anti-angiogenic therapy were mainly used in the second or further lines. PD-1-targeting inhibitors included nivolumab, pembrolizumab, sintilimab, and toripalimab. The following chemotherapy regimens were included: SOX (days 1–14: twice daily S-1 40–60 mg + day 1 oxaliplatin 130 mg/m^2^), DCF (cisplatin 75 mg/m^2^, docetaxel 75 mg/m^2^ + fluorouracil 750 mg/m^2^/day), and XELOX (days 1–14: twice daily capecitabine 1,000 mg/m² for each cycle + day 1 intravenous oxaliplatin 130 mg/m² for each cycle). Anti-angiogenic agents included either small-molecule tyrosine kinase inhibitors (TKIs) (apatinib) or monoclonal antibodies (bevacizumab). The therapy was selected based on the patient’s preferences and clinical status.

### Development of a prognostic model

Univariate Cox analysis was used to select potential risk factors for survival. <ca>A</ca> *p*-value <0.05 was considered statistically significant. After the potential risk factors were selected, we performed multivariate analyses with three selection procedures (forward, backward, and stepwise) to select the best-fit model. Based on the results of multivariate Cox proportional hazards, statistically significant variables (*p* < 0.05) were included in the nomogram to predict the 1- and 2-year survival with immunotherapy treatment.

### Model validation and clinical use

The predictive performance of the nomogram model was assessed by the concordance index (C-index) and calibration. The C-index was used to assess discrimination. <ca>A</ca> C-index of 0.5 indicates no predictive discrimination, while 1.0 indicates a perfect separation of patients with different outcomes. Calibration plots were used to compare the nomogram-predicted probability and the actual outcome. Kaplan–Meier curves were plotted over the median nomogram-predicted score for further evaluation. Finally, the net benefit of the model was assessed using decision curve analysis (DCA).

### Statistical analysis

Continuous variables are described by medians with interquartile ranges (IQRs) and count data by proportions. The OS was calculated from the first immunotherapy administration date until death due to any cause. Survival curves were depicted using the KM method and compared using the log-rank test. Cox regressions were used to determine prognostic indexes. The nomogram was plotted based on the multivariate analysis results using the “rms26” R package. The C-index was calculated using the “Hmisc” R package. Bootstraps with 1,000 resamples were used to draw calibration curves to evaluate the fitting degree of the nomograms. Calibration plots were evaluated by the “rms” R package. The DCA was performed using the “ggDCA” R package. Statistical analyses were performed in R (v. 4.1.2), and a *p*-value <0.05 was considered statistically significant.

## Results

### Patient characteristics

The baseline clinical characteristics of patients in the training (*n* = 186) and validation (*n* = 80) cohorts are described in **Table 1**. In the training set, the median OS (mOS) was 13.25 months (95% CI: 11.30–15.19), and the 1- and 2-year survival rates were 46.8% and 15.1%, respectively. Among the training cohort cases, 132 were men and 54 women, with a median age of 59. Additionally, 146 patients (78.5%) had ECOG PS of 0–1, and the remaining had ECOG PS of 2. Body/fundus was the most common tumor location (*n* = 116, 62.4%). Poor differentiation accounted for 54.8% (*n* = 102) and 82 patients (44.1%) received gastrectomy. Moreover, 102 (54.8%) patients received immunotherapy as the first-line treatment, while the rest received immunotherapy as second or further lines. Furthermore, 65.6% of the patients suffered more than one organ metastases. According to the ROC analyses, the optimal cutoffs by the NLR, PLR, MLR, and dNLR for predicting the 6-month survival were 3.23, 0.29, 0.38, and 2.07, respectively. The good LIPI group included 45.7% of the patients based on the dNLR cutoff and the ULN of LDH of 250 U/L. The baseline characteristics were similar between the training and validation cohorts.

**Table 1:** Patient characteristics of the training cohort and the validation cohort.

Characteristics	Level	Training (*n* = 186)	Validation (*n* = 80)	*p*-value
Gender (%)	Female	54 (29.0)	25 (31.2)	0.828
	Male	132 (71.0)	55 (68.8)	
Age (%)	<59	89 (47.8)	42 (52.5)	0.574
	≥59	97 (52.2)	38 (47.5)	
PD-L1 (%)	Negative	91 (48.9)	46 (57.5)	0.079
	Positive	46 (24.7)	23 (28.7)	
	Unknown	49 (26.3)	11 (13.8)	
ECOG PS (%)	0–1	146 (78.5)	66 (82.5)	0.563
	2	40 (21.5)	14 (17.5)	
Tumor location (%)	Body/fundus	116 (62.4)	49 (61.3)	0.818
	Cardia	43 (23.1)	21 (26.2)	
	Pylorus	27 (14.5)	10 (12.5)	
Differentiation (%)	Moderately/well	84 (45.2)	41 (51.2)	0.436
	Poorly	102 (54.8)	39 (48.8)	
Surgery (%)	No	104 (55.9)	37 (46.2)	0.189
	Yes	82 (44.1)	43 (53.8)	
Number of metastatic organs (%)	<2	64 (34.4)	28 (35.0)	1.000
	≥2	122 (65.6)	52 (65.0)	
Liver metastases (%)	No	121 (65.1)	42 (52.5)	0.073
	Yes	65 (34.9)	38 (47.5)	
Treatment line (%)	First	102 (54.8)	36 (45.0)	0.181
	Others	84 (45.2)	44 (55.0)	
Treatment type (%)	Anti-PD-1 monotherapy	9 (4.8)	7 (8.8)	0.309
	Anti-PD-1 plus anti-angiogenic therapy	30 (16.1)	16 (20.0)	
	Anti-PD-1 plus chemotherapy	147 (79.0)	57 (71.2)	
Hemoglobin (%)	<110	93 (50.0)	48 (60.0)	0.172
	≥110	93 (50.0)	32 (40.0)	
NLR (%)	<3.23	108 (58.1)	39 (48.8)	0.205
	≥3.23	78 (41.9)	41 (51.2)	
PLR (%)	<139.41	72 (38.7)	30 (37.5)	0.961
	≥139.41	114 (61.3)	50 (62.5)	
LIPI group (%)	Good LIPI	85 (45.7)	34 (42.5)	0.729
	Intermediate/poor LIPI	101 (54.3)	46 (57.5)	
LDH (%)	<250	86 (46.2)	31 (38.8)	0.321
	≥250	100 (53.8)	49 (61.3)	
Baseline CEA (%)	<5	97 (52.2)	36 (45.0)	0.349
	≥5	89 (47.8)	44 (55.0)	
Baseline CA 19-9 (%)	<37	103 (55.4)	47 (58.8)	0.708
	≥37	83 (44.6)	33 (41.2)	
MLR (%)	<0.38	104 (55.9)	43 (53.8)	0.848
	≥0.38	82 (44.1)	37 (46.2)	

ECOG PS, Eastern Cooperative Oncology Group performance score; PD-L1, programmed death-ligand 1; NLR, neutrophil-to-lymphocyte ratio; PLR, platelet-to-lymphocyte ratio; LIPI, lung immune prognostic index; CA 19-9, carbohydrate antigen 19-9; CEA, carcinoembryonic antigen; LDH, lactate dehydrogenase; MLR, monocyte-to-lymphocyte ratio.

### Development of the nomogram prognostic model

**Figure 1:**
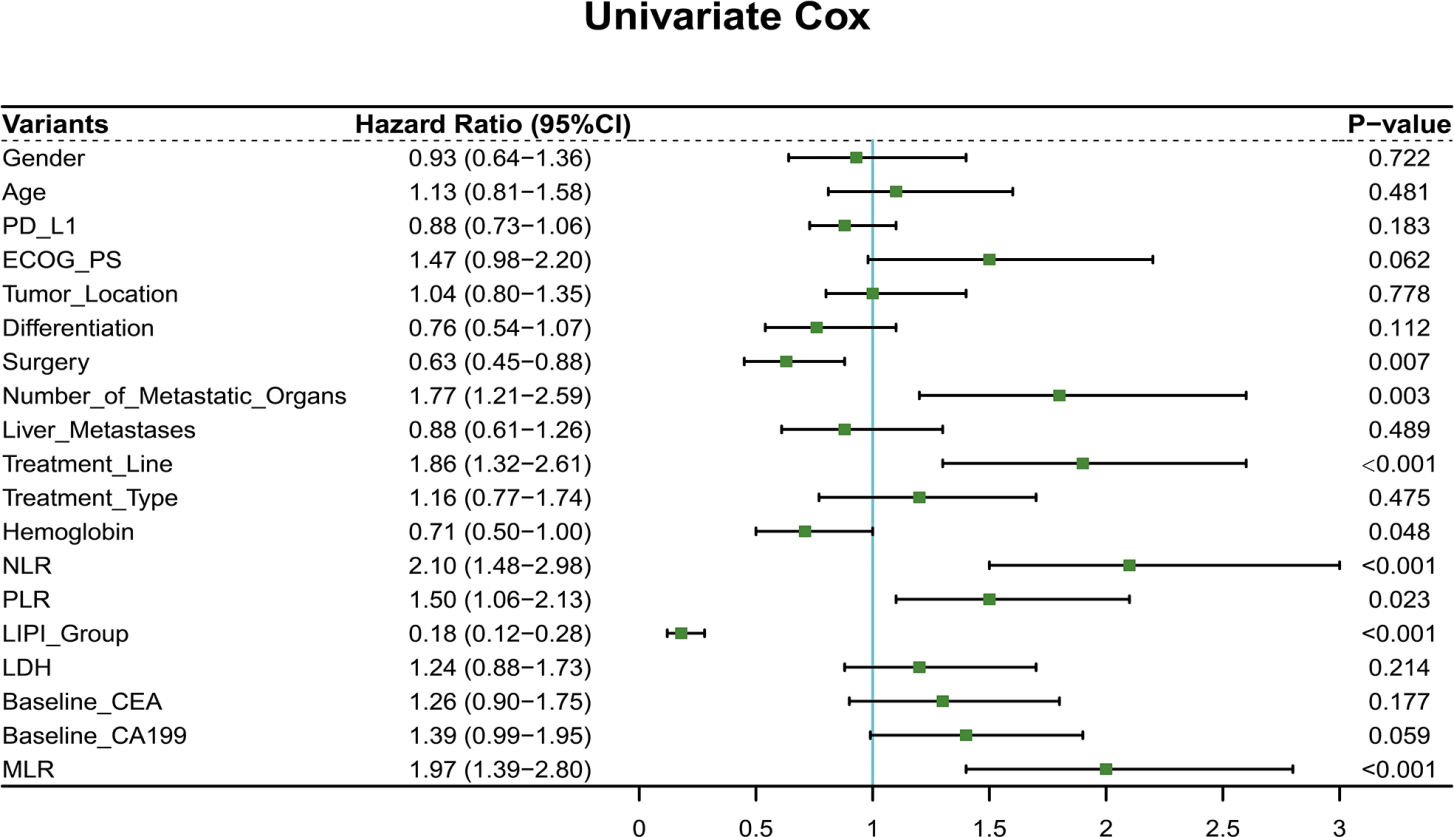
Univariate Cox regression of all features for overall survival in the training cohort.

**Figure 2:**
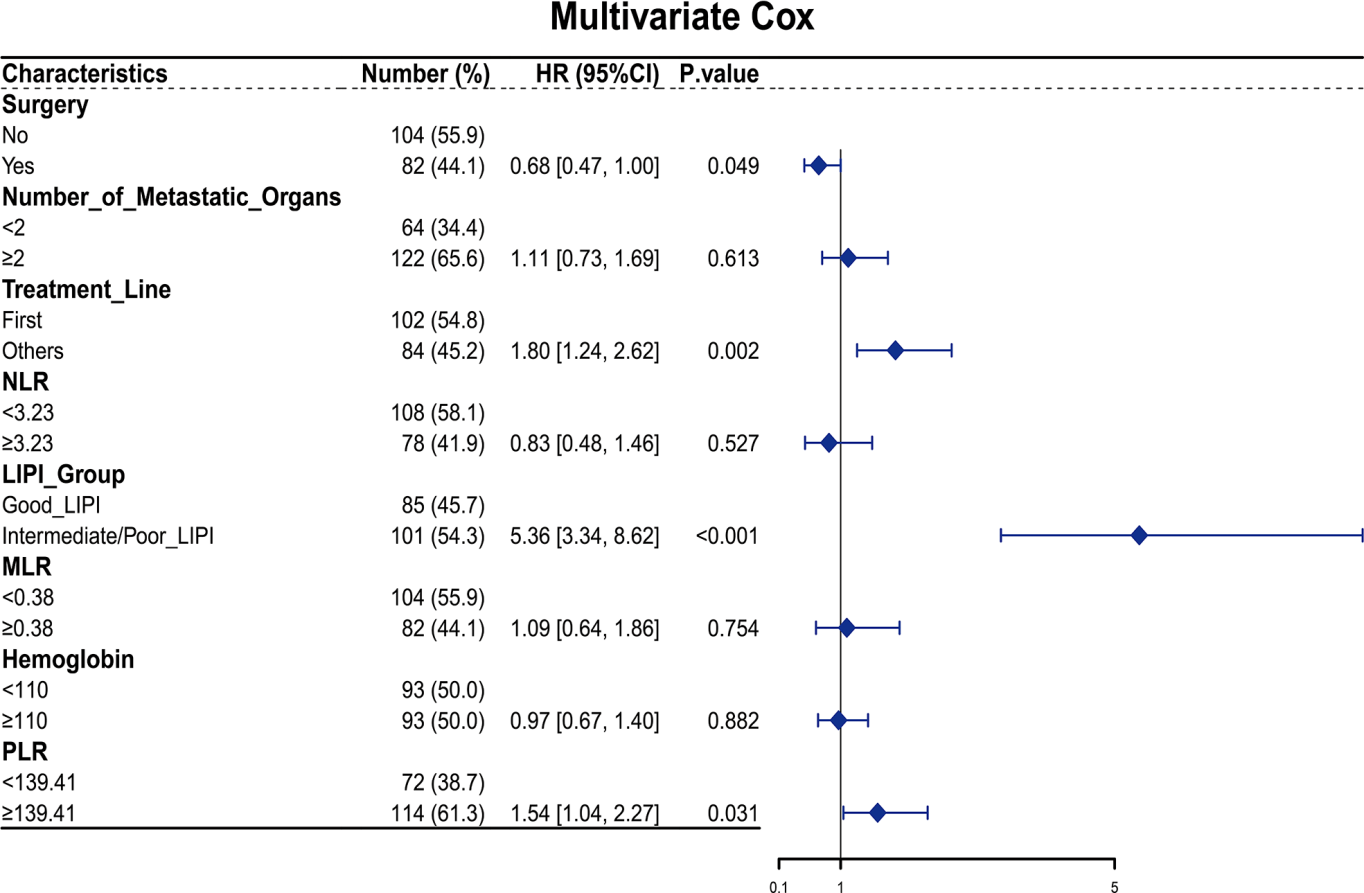
Multivariate Cox regression for overall survival in the training set.

Furthermore, 19 clinicopathological variables were analyzed for their association with the OS. In the univariable analysis, surgery (*p* = 0.007, HR = 0.63, 95% CI: 0.45–0.88), number of metastatic organs (*p* = 0.003, HR = 1.77, 95% CI: 1.21–2.59), treatment line (*p* < 0.001, HR = 1.86, 95% CI: 1.32–2.61), hemoglobin (*p* = 0.048, HR = 0.71, 95% CI: 0.50–1.00), NLR value (*p* < 0.001, HR = 2.10, 95% CI: 1.48–2.98), PLR value (*p* = 0.023, HR = 1.50, 95% CI: 1.06–2.13), LIPI group (*p* < 0.001, HR = 0.18, 95% CI: 0.12–0.28), and MLR (*p* < 0.001, HR = 1.97, 95% CI: 1.39–2.80) were associated with the OS (*p* < 0.05) (**Figure 1**). Next, the multivariable Cox regression model included eight variables based on the univariable results. The multivariate analysis demonstrated that surgery (*p* = 0.049, HR = 0.68, 95% CI: 0.47–1.00), treatment line (*p* = 0.002, HR = 1.80, 95% CI: 1.24–2.62), LIPI group (*p* < 0.001, HR = 5.36, 95% CI: 3.34–8.62), and PLR (*p* = 0.031, HR = 1.54, 95% CI: 1.04–2.27) were independent risk factors for the OS (**Figure 2**).

### Validation performance of the nomogram

**Figure 3:**
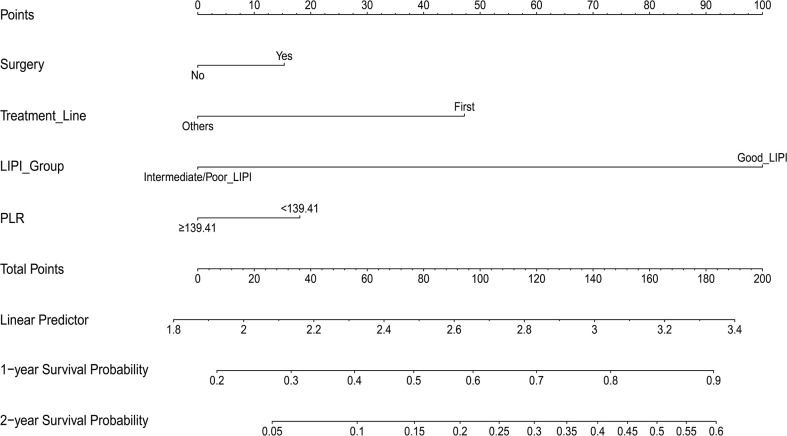
Nomogram for predicting 1- and 2-year survival in patients with metastatic gastric cancer.

**Figure 4:**
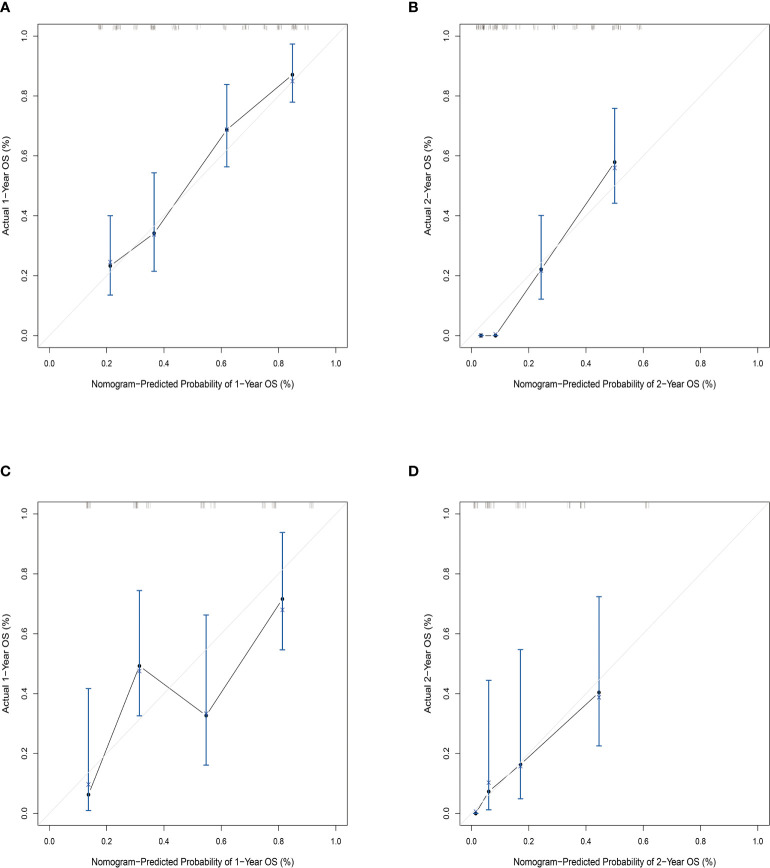
Calibration plot of the nomogram model comparing predicted probabilities with actual 1- and 2-year survival rates. **(A)** Calibration plot of 1-year survival prediction in the training set nomogram; **(B)** calibration plot of 2-year survival prediction in the training set nomogram; **(C)** calibration plot of 1-year survival prediction in the validation set nomogram; **(D)** calibration plot of 2-year survival prediction in the validation set nomogram. The c-index in the training set is 0.745, and in the validation set, it is 0.713.

Then, the prognostic nomogram was constructed based on the above multivariate analysis (**Figure 3**). The C-index obtained for the nomogram by bootstrap resampling in the training set was 0.745. The calibration curves for the probability of survival at 1 (**Figure 4A**) and 2 years (**Figure 4B**) demonstrated good agreement between nomogram predictions and actual observations. In the validation dataset, the calibration plots demonstrated good performance of the nomogram models (**Figures 4C, D**), and the C-index for OS prediction was 0.713.

### Survival curves based on the nomograms and DCA

**Figure 5:**
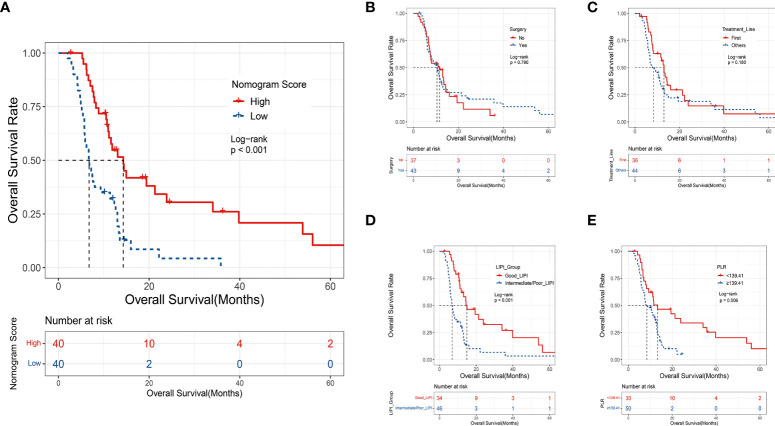
**(A)** The KM curve of the validation set based on the median score of the nomogram model; **(B)** the KM curve of the validation set based on surgery history; **(C)** the KM curve of the validation set based on treatment line; **(D)** the KM curve of the validation set based on the LIPI group; **(E)** the KM curve of the validation set based on the PLR group.

**Figure 6:**
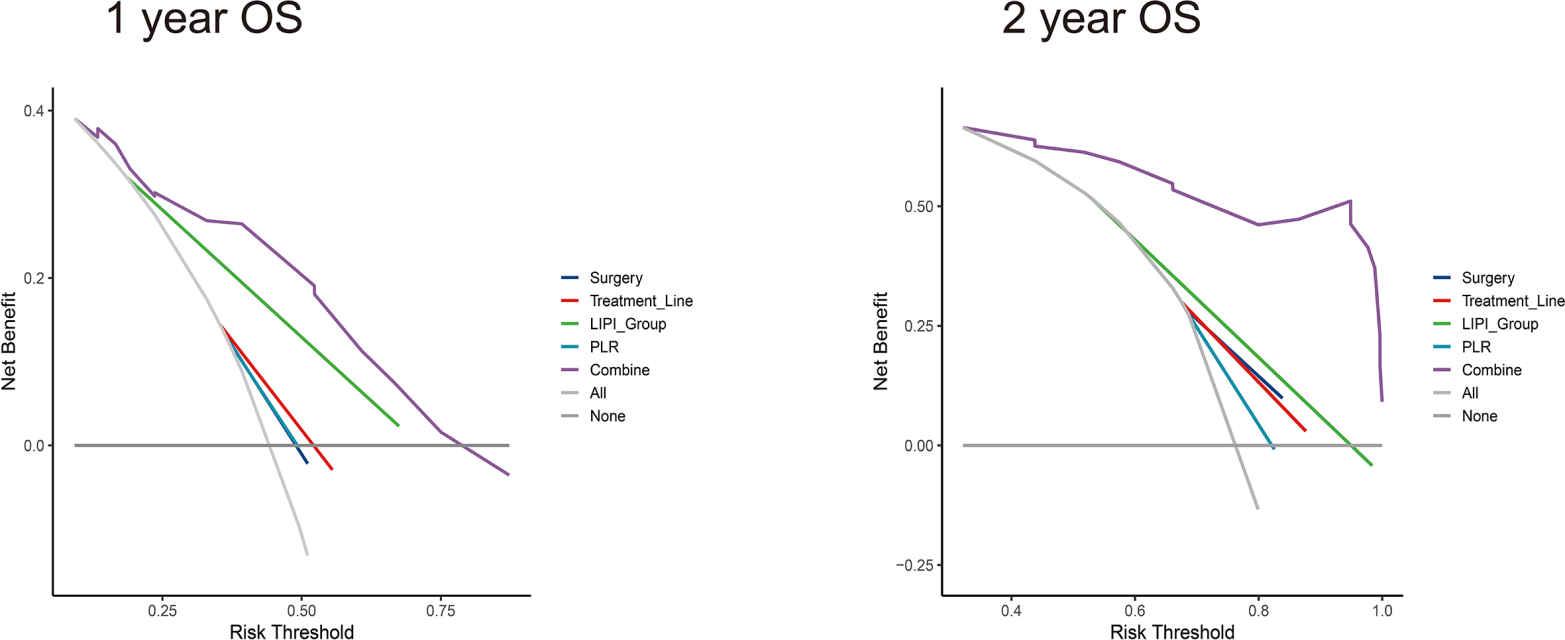
Decision curve analysis (DCA) of nomograms for the training set to predict 1- and 2-year survival. (Left) DCA for 1-year survival prediction in the nomogram; (right) DCA for 2-year survival prediction in the nomogram.

The median nomogram-predicted score was 79.62. The survival curves stratified by the median nomogram-predicted score are shown in **Figure 5A**. In the validation cohort, patients with a nomogram-predicted score higher than 79.62 presented substantially better survival than those with a nomogram score lower than 79.62 (*p* < 0.001). We also plotted the KM curve forthe four individual factors in the validation cohort (**Figures 5B–E**). Surgery history and treatment line were not associated with the OS (*p* = 0.790 and 0.180, respectively). These results suggested that the nomogram was a better predictor for the OS of patients than single factors. DCA was also used to validate the clinical utility of the model based on the net benefit. The combined predictive models showed better clinical utility than the predictive value of any single variable (**Figure 6**).

## Discussion

Due to the heterogeneity of GC, its prognosis is affected by various factors. The outcomes of CheckMate 649 (26) and ATTRACTION-4 (27) paved the way for immunotherapy for MGC patients, especially with PD-L1 CPS ≥5. Many biomarkers have been proposed as prognostic indexes for immunotherapy patients. However, the relationship between PD-L1 expression and survival in immunotherapy-based clinical trials for GC is not consistent (10, 27, 28). High TBM (TMB-H) has been proposed as a predictive biomarker for the response to immune checkpoint blockade (ICB) for many cancers (29). However, few studies have demonstrated that the TMB-H is associated with a better response in GC patients. Although the FDA has approved the PD-1 inhibitor pembrolizumab for solid tumors in patients harboring MSI-H/dMMR, the incidence of MSI-H GC in Asians is commonly below 10% of all GC cases, lower than most rates reported in the Western population (30). Therefore, these well-known biomarkers, TMB and MSI-H, do not cover all GC patients but are routinely used in clinical practice. On the other hand, the predictive value of blood parameters, such as the system inflammation index and the easily taken and continent biomarkers, has been explored for GC patients under adjuvant therapy or palliative chemotherapy (15, 31, 32). Hemoglobin (18) or other derived inflammation indexes (dNLR) (33) and serum tumor markers (34) can also be associated with the outcomes of chemotoxicity agents in GC. Nevertheless, no studies have combined clinical characteristics and these blood markers to predict the survival of GC patients receiving anti-PD-1 therapy. Hence, in the present study, we used data from two medical centers and a real-world study to establish and validate a prognostic nomogram based on clinicopathological characteristics and other variables.

To the best of our knowledge, this is the first nomogram for predicting the survival of MGC patients under immunotherapy. We identified four independent prognostic factors (surgery history, treatment line, LIPI, and PLR) that were further included in the nomogram based on their statistical significance. The nomogram presented good applicability in the training (C-index = 0.745) and validation (C-index = 0.713) sets. Furthermore, based on the median nomogram-predicted score (79.62), the MGC patients in the validation cohort presented considerable OS risk profiles with clearly defined risk groups. Sun Young Kim and colleagues constructed a similar nomogram for the 1- or 2-year survival after patients received chemotherapy (24) with a C-index of 0.656 for the baseline and 0.718 for the chemo response-based nomogram. However, this nomogram did not include system inflammation indexes and tumor makers. Tai Ma et al. also developed a nomogram for predicting survival in MGC patients who underwent palliative gastrectomy (35). Age, tumor size, location, grade, T stage, N stage, metastatic site, gastrectomy scope, number of examined lymph nodes, chemotherapy, and radiotherapy were included as variables in this nomogram; the C-index values of the development and validation datasets were 0.701 and 0.699, respectively. Nevertheless, their prognostic factors were different from the variables used in our current study. Other studies developed a prognostic risk stratification nomogram for GC patients after radical gastrectomy (36–38). Here, we constructed for the first time a nomogram for immunotherapy-based therapy for MGC. The nomogram showed good accuracy in the external validation, demonstrating its potential for clinical use.

Nomograms are more accurate than conventional markers for predicting the prognosis of some cancers (39). Consistently, we demonstrated that the combined model (four variables) presented better clinical value in predicting the 1- and 2-year survival than any single variable based on the DCA. Several variables have prognostic value, as reported in previous studies. The LIPI was first proposed by Mezquita et al. (40) and showed prognostic value in NSCLC patients treated with cytotoxic chemotherapy or targeted therapy (41, 42). In our previous study, we found that the LIPI correlates with better outcomes for MGC patients in the anti-PD-1-treated group but not in the chemotherapy-treated group (manuscript under review). The prognostic role of PLR was previously demonstrated for GC in a meta-analysis (17). In the subgroup analysis of the CheckMate 649 study (43), patients with previous surgery had a median OS of 15.4 months compared with 13.6 months in the no-surgery group. Although many prognostic variables have been explored in other studies, combining these four variables improved the prognostic value of the model. PD-L1 was excluded from the nomogram due to no significant difference (*p* = 0.183). Of note, PD-L1 expression can be used to predict the efficacy of monoimmunotherapy, but the prognostic value of PD-L1 inhibitors with chemotherapy remains unconfirmed.

Our current study also has some limitations, including biases from its retrospective nature. First, the nomogram was created using data from two medical centers with independent operating systems in the same city. Second, the population of the validation set was limited. Thus, large populations are required to verify the predictive performance of the nomogram. Third, the standard of care of the second line remains platinum, taxane, and irinotecan for most MGC cases. Here, we had some exploration treatments such as immunotherapy plus anti-angiogenic agents in the second or further lines.

In summary, we used four variables to construct a reliable prognostic nomogram for predicting the survival of MGC patients treated with immunotherapy. Finally, further studies are required to determine whether it can be applied to other patient groups.

## Data availability statement

The raw data supporting the conclusions of this article will be made available by the authors, without undue reservation.

## Ethics statement

The studies involving human participants were reviewed and approved by the Ethics Committee of PLAGH. The patients/participants provided their written informed consent to participate in this study.

## Author contributions

MG, NQ, YZ, and LW were responsible for the data collection. GD and ZW supervised the study. QF and LW contributed to the data analysis. All authors contributed to the article and approved the submitted version.

## Conflict of interest

Author LW and QF were employed by the company Genetron Health (Beijing) Co. Ltd.

The remaining authors declare that the research was conducted in the absence of any commercial or financial relationships that could be construed as a potential conflict of interest.

## Publisher’s note

All claims expressed in this article are solely those of the authors and do not necessarily represent those of their affiliated organizations, or those of the publisher, the editors and the reviewers. Any product that may be evaluated in this article, or claim that may be made by its manufacturer, is not guaranteed or endorsed by the publisher.
