# Supplementary figures and images for: Construction of a nomogram to predict the survival of metastatic gastric cancer patients that received immunotherapy
Source: Front Immunol. 2022 Sep 26;13:950868. doi: 10.3389/fimmu.2022.950868 (PMC9549034; doi:10.3389/fimmu.2022.950868)

Overall Survival Rate

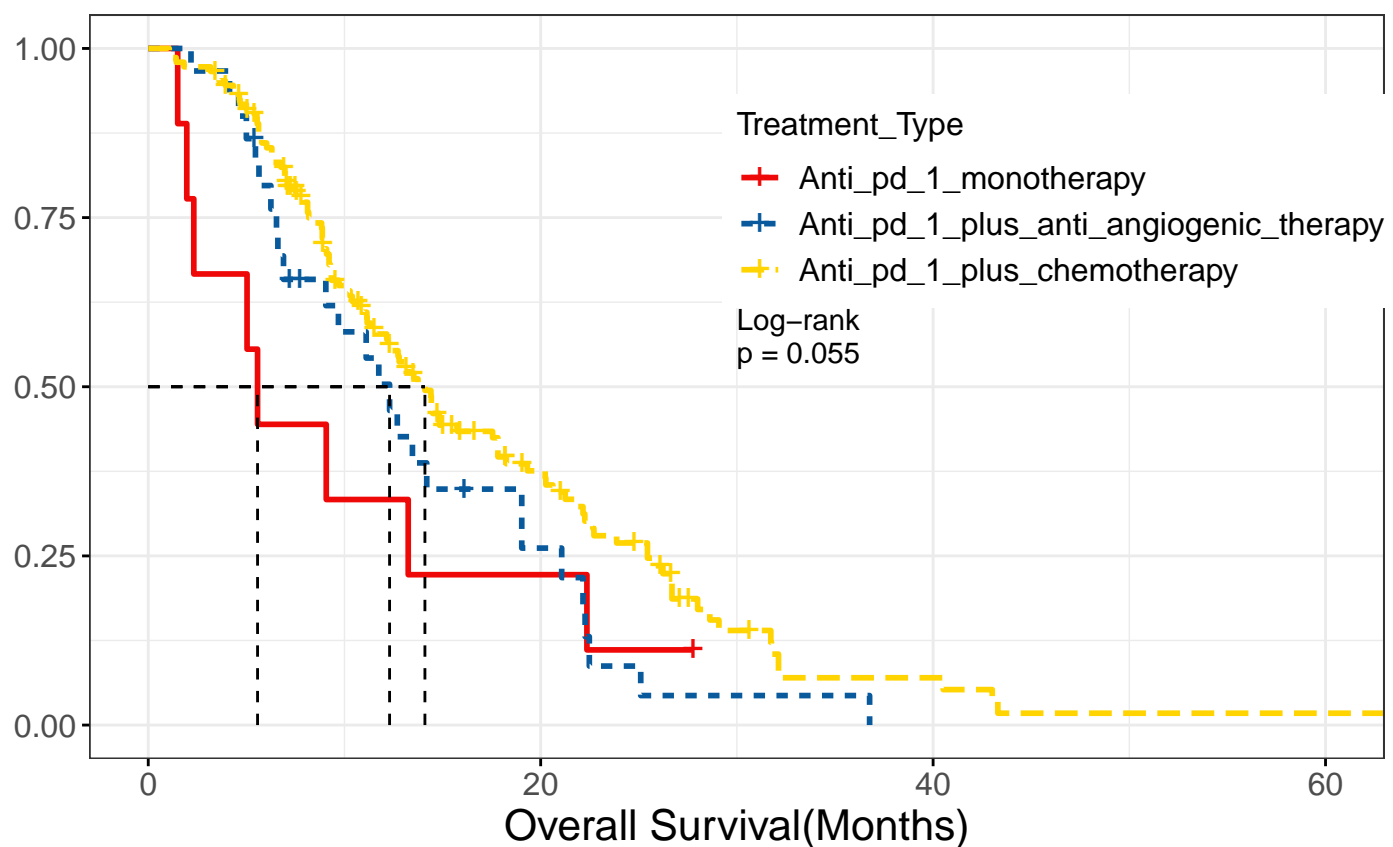

Treatment\_Type

Number at risk

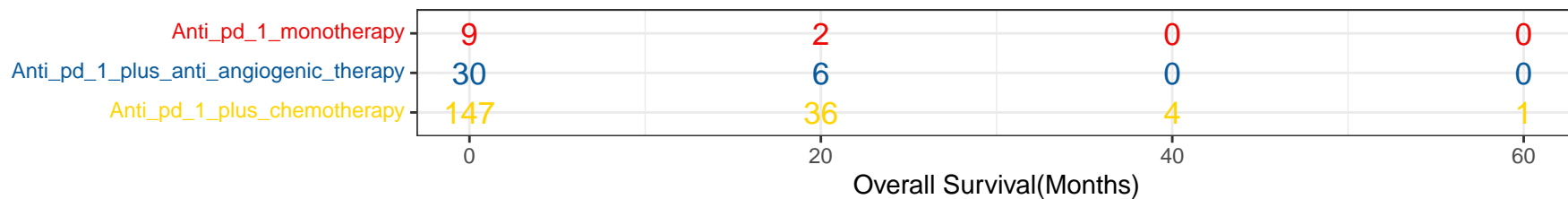

Supplement: Supplementary file 1 [file Image_1.pdf]

Overall Survival Rate

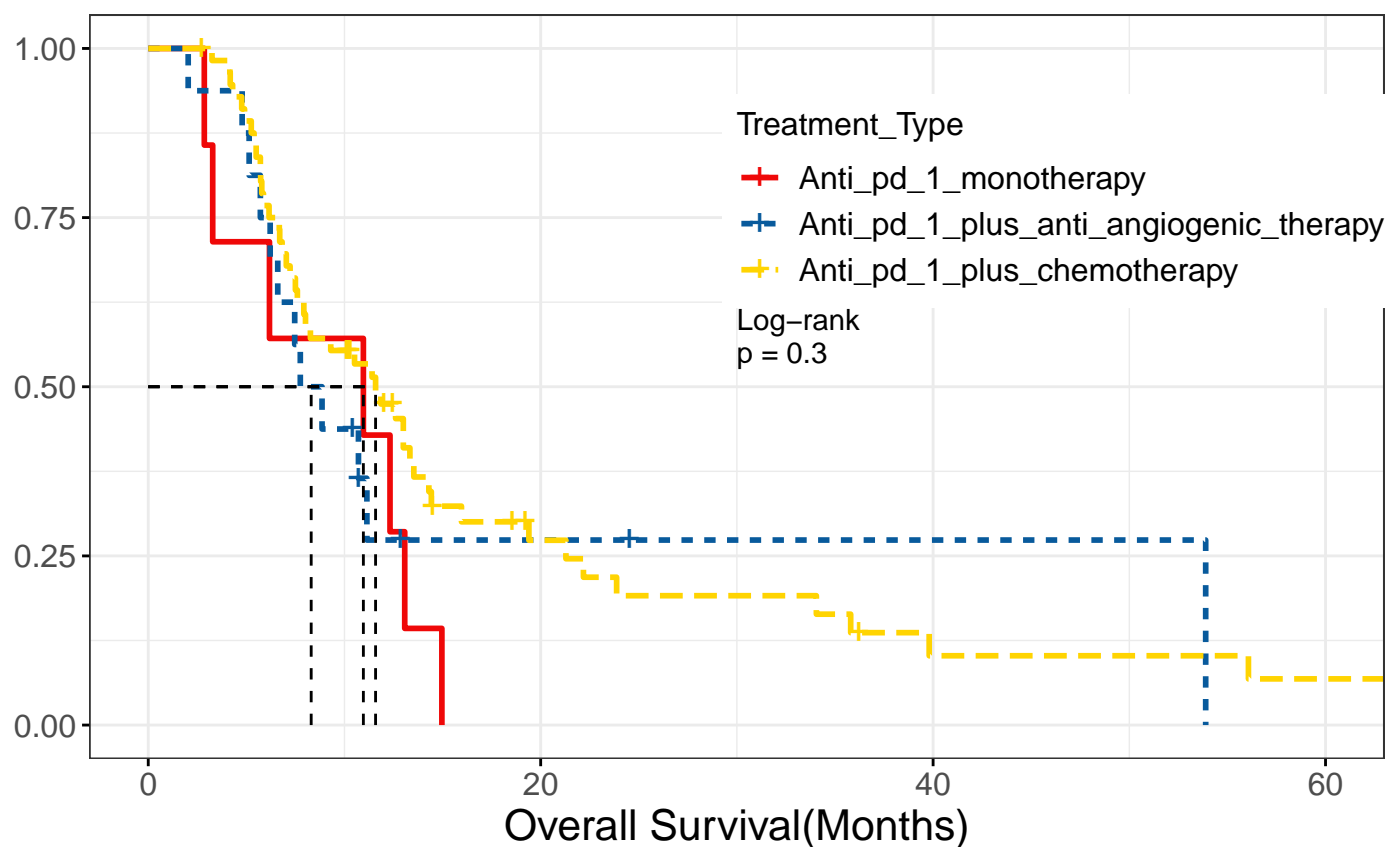

Number at risk

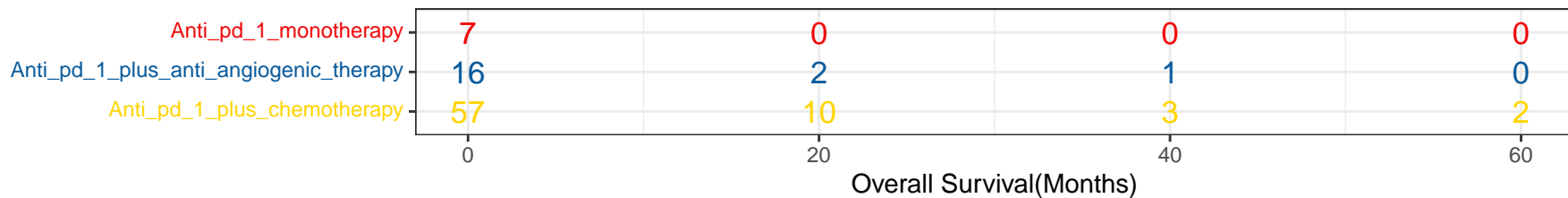

Supplement: Supplementary file 2 [file Image_2.pdf]
